# Effect of Load on the Corrosion Behavior of Friction Stir Welded AA 7075-T6 Aluminum Alloy

**DOI:** 10.3390/ma13112600

**Published:** 2020-06-07

**Authors:** Marina Cabrini, Sara Bocchi, Gianluca D’Urso, Claudio Giardini, Sergio Lorenzi, Cristian Testa, Tommaso Pastore

**Affiliations:** 1 Department of Engineering and Applied Sciences, University of Bergamo, 24044 Dalmine (BG), Italy; sergio.lorenzi@unibg.it (S.L.); cristian.testa@unibg.it (C.T.); tommaso.pastore@unibg.it (T.P.); 2 National Interuniversity Consortium of Materials Science and Technology (INSTM) Research unit of Bergamo, 24044 Dalmine (BG), Italy; 3 Center for Colloid and Surface Science (CSGI) Research unit of Bergamo, 24044 Dalmine (BG), Italy; 4 Department of Management, Information and Production Engineering, University of Bergamo, 24044 Dalmine (BG), Italy; sara.bocchi@unibg.it (S.B.); gianluca.d-urso@unibg.it (G.D.); claudio.giardini@unibg.it (C.G.)

**Keywords:** corrosion, stress-corrosion cracking, friction stir welding (FSW), AA7075

## Abstract

The paper focuses on the corrosion behavior of aluminum joints made by friction stir welding as a function of loading conditions. A four-points bend-beam test, constant loading test, and slow strain-rate test were carried out on AA 7075-T6 alloy in aerated NaCl 35g/L solution at room temperature monitoring the free corrosion potential. The penetration depth of the intergranular attack was deeper after the four-point bent-beam tests compared to all the other testing techniques. Preferential dissolution along the grain boundaries was found in the heat-affected zone and the attack follows the elongated grains structure along the rolling direction. However, no stress-corrosion cracking phenomena were detected. No relevant stress corrosion cracking (SCC) crack embryos propagation was noticed under uniaxial tensile tests—both constant loading and slow strain-rate tests—manly due to the high dissolution rate occurring at the crack tip which promoted premature shear ruptures.

## 1. Introduction

Friction stir welding (FSW) is a recent solid-state joining technique developed by the Welding Institute [1,2]. This technique has promising applications on difficult-to-weld alloys, i.e., age-hardening aluminum alloys [3,4]. FSW technique has been successfully applied to Al-Cu 2XXX series [5,6], Al-Si-Mg 6XXX [7,8], and Al-Zn-Mg 7XXX series alloys [9,10].

The 7XXX series aluminum alloys are widely used in aircraft and automotive industry due to the high tensile strength on weight ratio. The corrosion resistance of the alloy is significantly affected by the presence of Zn and Mg, which could induce several corrosion phenomena, such as localized corrosion such as pitting [11,12,13], intergranular [14,15], and stress-corrosion cracking [16,17].

The corrosion behavior of such alloys is strictly related to the temper. Several authors confirm that the 7XXX alloy exhibits SCC susceptibility in peak-aged conditions (T6 temper). However, over-aging (T7) or retrogression and re-aging (RRA) treatments are not critical compared to SCC [18,19]. Even if FSW is a solid-state joining technique, it is well known that it implies deep modifications to the alloy’s microstructure and precipitates’ distribution in the welding zones [20,21,22,23]. Therefore, this process can significantly affect the corrosion resistance and SCC [24,25].

The aim of this work is the study of the SCC susceptibility of AA 7075 T6 FSW butt joints by means of constant deformation tests with a four-point bending device (4PBD), uniaxial constant loading (CL) and slow strain-rate (SSR) tests in NaCl 35g/L aerated solutions at room temperature. During the tests, the open circuit potential (OCP) was monitored. The corrosion morphology was observed after exposure by means of the optical microscope and the scanning electron microscope (SEM) equipped with an energy dispersive X-ray spectroscopy device (EDX).

## 2. Materials and Methods

### 2.1. Welds

Sheets with a thickness equal to 4 mm were considered. Table 1 shows the chemical composition of the AA 7075 T6 alloy. Sheets of 200 mm × 80 mm were welded by using a custom-made tool designed with a smooth plane shoulder (16 mm diameter) coupled with a frustum cone-shaped pin (maximum and minimum pin diameters were 6 mm and 4 mm, pin height was 3.8 mm). The rotational speed of the tool (S) was 1500 rpm and feed rate (F) was 10 mm/min. The set-up of the welding procedure was reported in a previous work [26]. The mechanical properties of the base materials and the welded joints are reported in Table 2. Good reproducibility was observed in terms of tensile strength of the FSW joints. Owing to non-uniform strain distribution, it was not possible to calculate the yield strength of the welded specimens.

### 2.2. Metallographic Analysis

The microstructure was revealed through Keller’s etchant after grinding by means of emery papers up to 4000 grit, then polishing with 0.1 µm alumina aqueous suspension and finally observed with Nikon (Eclipse MA100N, Tokyo, Japan) optical microscope and SEM (Zeiss EVO 50, Zeiss, Oberkochen, Germany) Oxford x-act sdd (silicon drift detector, nitrogen free) EDX equipment type model (Oxford Instruments, High Wycombe, UK).

### 2.3. OCP Monitoring

OCP was measured on the three different zones of FSW butt welds, i.e., the nugget, the thermomechanical affected zone/heat-affected zone (TMAZ/HAZ—it was not possible to separate the two zones) and the base metal. The specimens were cold-mounted in epoxy resin with an embedded wire to allow electrochemical measurements. A 20 mm × 20 mm area was grounded with emery paper and then polished with colloidal alumina up to 0.1 μm. After polishing, the specimens were stored at room temperature (23 °C) with relative humidity in the range of 25–30% for 48 h before the immersion, to allow the formation of the natural protective film. The specimens were dipped in different testing cells filled with aerated 35 g/L NaCl solution (Carlo Erba RPA reagent, Cornaredo, Italy). The OCP was measured by using a saturated calomel electrode (SCE E = +0.240 V vs Standard Hydrogen Electrode, Amel Instrument, Milano, Italy) positioned close to the surface of the specimens to reduce the ohmic drop in the electrolyte during electrochemical tests.

### 2.4. Four-Point Bending Tests 

**Figure 1:**
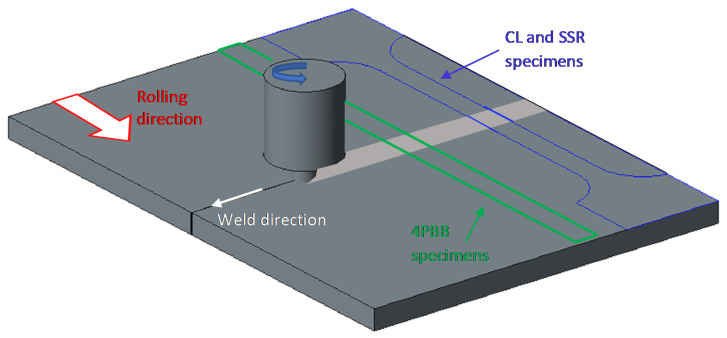
Schematic representation of specimens obtained by means of friction stir welding (FSW) welded sheet (draw not in scale).

Four-point bending (4PBB) tests have been performed according to ASTM G39 on 4 mm × 20 mm × 160 mm prismatic specimens obtained by FSW joints (Figure 1). The specimens were polished by emery papers up to 1000 grit and degreased in acetone in ultrasonic bath for 5 min. After polishing, the specimens were left in still air for 48 h to allow the formation of the protective film.

The specimens were positioned inside the bending device to attain a uniform tensile strain distribution over the welded surface. Four glass cylinders were used to avoid galvanic coupling between the stainless-steel specimen holder and the aluminum alloy. Four specimens were loaded up to 80% of the tensile strength of FSW joints derived by tensile tests (refer to Table 2). The other four specimens were dipped without loading (unloaded condition). Tests have been performed on four specimens for each condition (loaded and unloaded). All the specimens were exposed in a cell filled with about 30 L water, and 35 g/L sodium chloride (Carlo Erba RPS analytical grade reagents, Cornaredo, Italy) for 1500 h. To avoid the contamination of the testing solution by corrosion products of aluminum during long-term exposures, the test solution was refreshed after 150 h and 500 h. The amount of water that was substituted was equal to 10 liters. During the procedure, the specimens were always left well below the waterline. The OCP was measured during the exposure. More details about the experimental set-up are reported in a previous work [27].

At the end of the long-term exposure tests, the specimens were unloaded, washed in distilled water, and then rinsed with acetone in an ultrasonic bath. The surfaces were then observed by means of optical microscopy up to 600 times magnification and SEM equipped with EDX.

### 2.5. Constant Load Tests 

**Figure 2:**
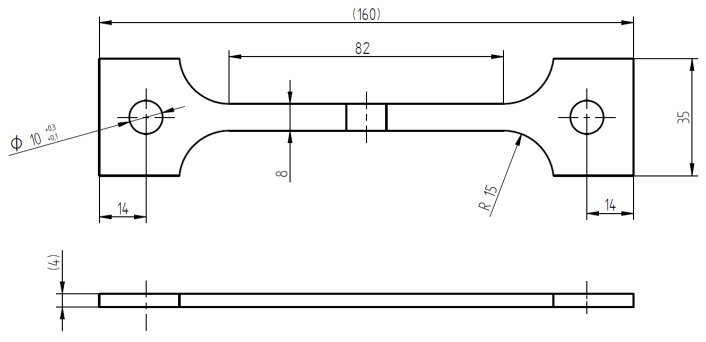
CL and SSR specimen: the weld is in the center of the gouge length, the thermomechanical affected zone/heat-affected zone (TMAZ/HAZ) extension is 40 mm for each side.

**Figure 3:**
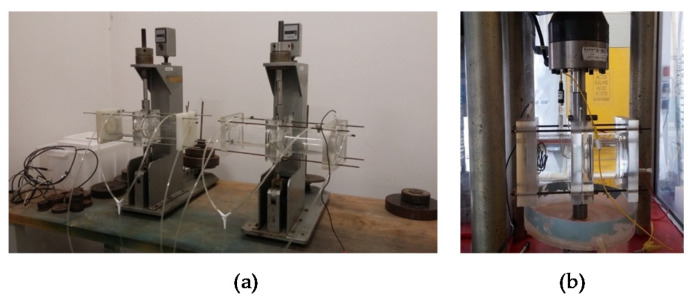
CL test device (**a**) and SSR test device (**b**).

CL tests were carried out on a specimen of 4 mm × 8 mm × 82 mm gage length, with the weld positioned at the center of each specimen (Figure 1). The size of the specimens is reported in Figure 2. The specimen was settled in a double compartment cell (Figure 3a). All the compartments were maintained at room temperature filled with aerated NaCl 35 g/L solution (Carlo Erba RPS analytical grade reagents, Cornaredo, Italy). Water recirculation in the cell was granted during the tests. The OCP of the specimens was continuously monitored using a SCE positioned close to the metal surface by means of a Lugging capillary.

The specimens in the cell were positioned into the CL machine (no loading), then they were dipped in the test solution. After 120 h of OCP monitoring, the specimens were loaded at 190 MPa, corresponding to 54% of the ultimate tensile strength (UTS) of the weld. This value is slightly lower than the elastic limit of the weld obtained by means of tensile tests. The specimens were left at this value for 350 h and the OCP was measured during all the period. Then, the applied load increased up to 279 MPa, corresponding to 80% of the UTS of the weld (homogeneous plastic deformation field). The OCP was measured until 720 h of exposure (one month). 

At the end of the test, the specimens were unloaded and observed under the optical microscope and under the SEM to analyze the corrosion morphology. Metallographic cross-section was also taken along the longitudinal direction of the specimen.

### 2.6. Slow Strain-Rate Tests

The SSR tests were carried out by using specimens of the same dimensions as the CL tests. The set-up of the tests is shown in Figure 3b. The tests were carried out on a 30 kN testing machine at displacement rates varying from 5 × 10^−7^ to 5 × 10^−3^ mm/s. A displacement rate of 8.2 × 10^−4^ mm/s was used in order to have a strain rate of 10^−6^ s^−1^ as average value on all the gage length. During the tests, the load and OCP were measured continuously. At the end of the tests the specimens were washed in distilled water and rinsed in acetone to permit the observation of the fracture surface under the SEM. Then the specimens were longitudinally sectioned for the metallographic observation.

## 3. Results

### 3.1. Metallographic Analysis 

**Figure 4:**
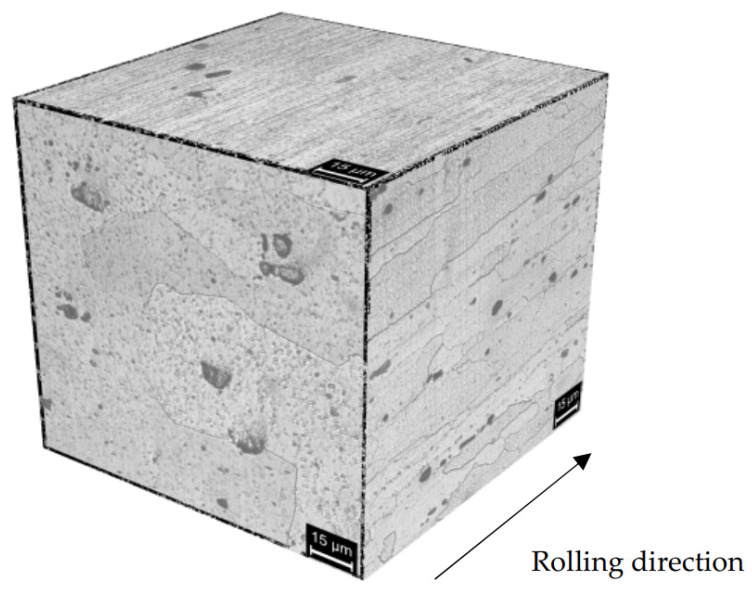
Optical image (Keller attack) of the alloy AA 7075 T6 (base material). Elongated grains along the rolling direction and the macro-precipitates are visible.

The microstructure of the AA 7075 T6 alloy has already been presented in [26]. Coarse precipitates along the grain boundaries (Figure 4) and micrometric precipitates within the grains of α-Al, oriented in the rolling direction [28], can be well demonstrated. The strengthening MgZn_2_ nanoprecipitates are not visible under the optic or SEM microscope, while macro-precipitates are copper and iron-rich phases as Al_2_CuMg and Al_7_Cu_2_Fe or zinc and magnesium rich phases, i.e., undissolved MgZn_2_ during alloy solution annealing treatment, or MgSi.

**Figure 5:**
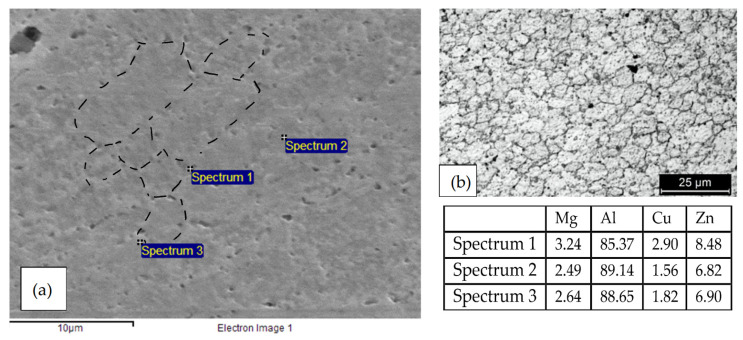
(**a**) SEM image of the nugget of the FWS 7075 alloy (no metallographic attack) the rounded recrystallized grains are visible owing to the precipitates of MgZn_2_ on their grain borders and EDX spectra in which is visible an enrichment of zinc and copper in the correspondence of the precipitates. In the image the grain border is demonstrated; (**b**) optical image of the nugget with metallographic attack (Keller’s reagent) that better demonstrated the rounded recrystallized grains.

In the nugget, re-crystalized fine equiaxed grain structure can be noticed. In addition, MgZn_2_ strengthening precipitates are dissolved (T6 artificial aging temper) into the supersaturated solid solution and reprecipitated after cooling (Figure 5). Venugopol et al. affirmed that the temperature obtained during the process is below the melting temperature of the alloy, but above the solutioning temperature, as demonstrated by the re-crystallization of the weld nugget and the redistribution of the precipitates [21]. For the same authors, the absence of fine precipitates in the weld nugget indicates that the cooling rates are such that larger precipitates could nucleate and grow but not the finer ones. In the TMAZ, the precipitates are quite random, and the coarsening of finer precipitates observed in base material can occur during welding [21].

**Figure 6:**
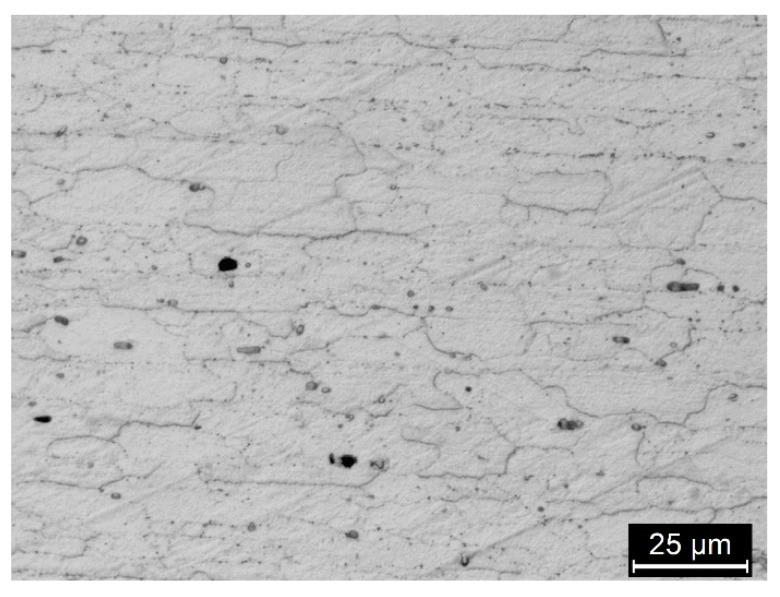
Optical image (Keller attack) of the HAZ of the FWS AA 7075 alloy. The grains appear elongated along the rolling direction; presence of macro-precipitates is evident.

The size of the precipitates at the grain boundaries of the HAZ (Figure 6) is unfortunately too small to permit their observation under the SEM, but many transmission electron microscope (TEM) observations were reported in the literature and they demonstrated that these smaller precipitates play a fundamental role in the intergranular corrosion and the SCC of these alloy series [18].

### 3.2. 4PBB Specimens

**Figure 7:**
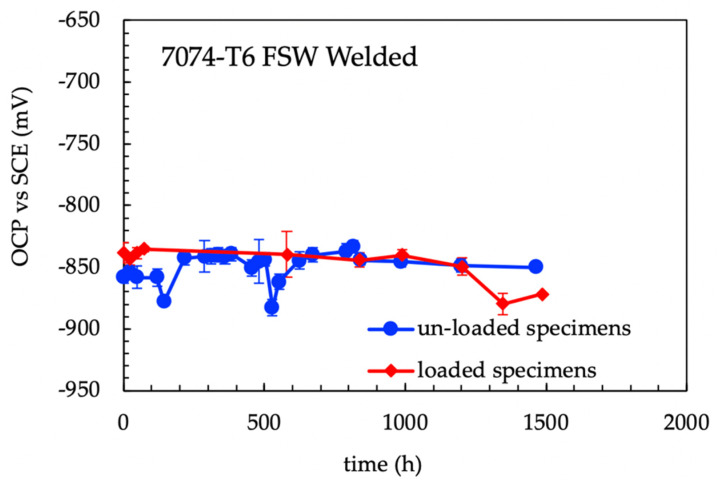
Open circuit potential (OCP) vs time for loaded and unloaded 4PBB specimens. The decreasing of the OCP of the specimens in the correspondence of the partial refresh of the test solution that breaks the aluminum oxide scale and exposes fresh metal surface to the aggressive solution.

**Figure 8:**
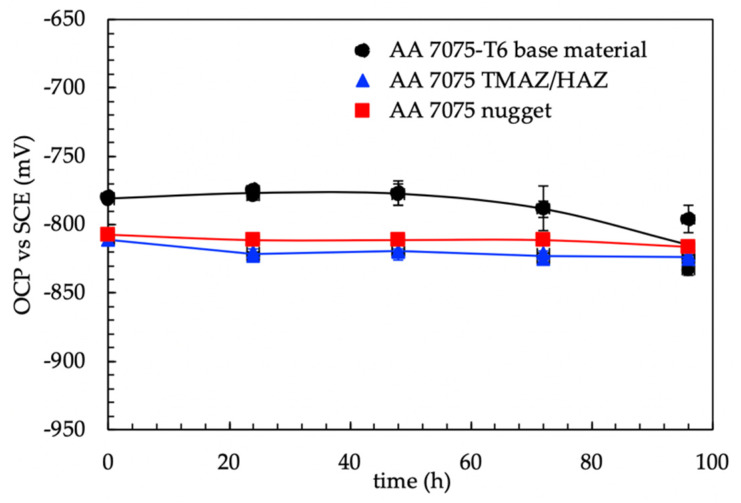
OCP vs time of the different zones (base materials, TMAZ and nugget) of the alloy AA7075 T6.

The OCP measurements (average values of four specimens) did not show any evident differences between 4PBB loaded and unloaded specimens (Figure 7). The corrosion potentials generally were in the range of −870–−840 mV vs SCE; the corrosion potential showed a sharp decrease during the solution refresh owing to the partial removal of non-adherent scale of corrosion products that exposes the corroded surface on the fresh solution; after this, the corrosion potential increases to the same potential values detected prior to the partial substitution of the testing solution. This effect was more marked for specimens without loading compared to loaded specimens. The corrosion potential of the loaded specimens decreases after 1300 h, then it increases again. Compared to the values obtained by the analysis of the three different zones (nugget, TMAZ/HAZ and base materials—Figure 8) similar results can be outlined in both the TMAZ/HAZ and the nugget. The base metal showed slightly nobler potential values.

**Figure 9:**
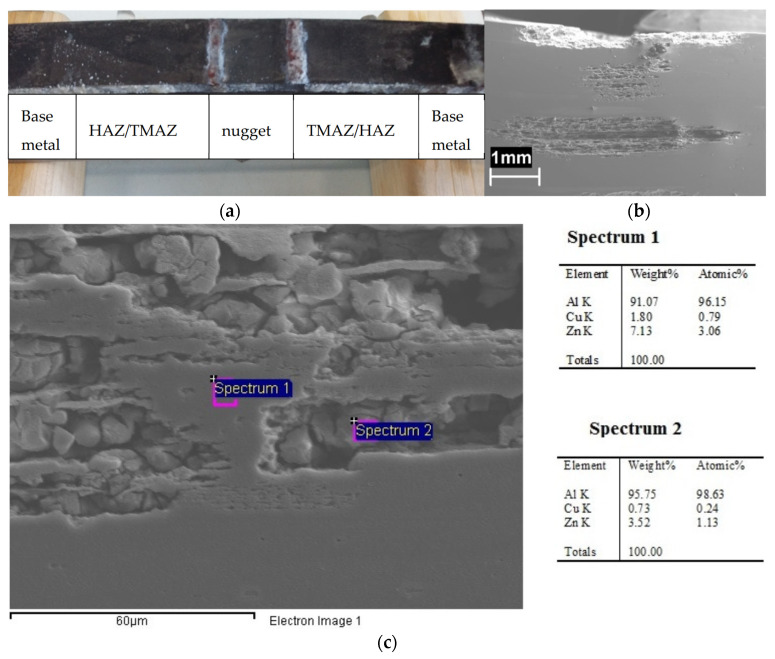
(**a**) Image of a 4PBB specimens after the test: an intense localized attack in the correspondence of the TMAZ and (**b**) SEM image of the metallographic longitudinal section of the loaded specimen in which is visible the deviation of the attack along the rolling direction, (**c**) particular of the attack and EDX analysis.

Figure 9a shows the corrosion morphology of the specimens at the end of the 4PBB tests. No differences in the corrosion morphology were observed between the loaded and unloaded specimens. The HAZ is preferentially corroded. The attack propagates along the grain boundaries, following the rolling direction (Figure 9b). The EDX analysis (Figure 9c) demonstrated the depletion of zinc and copper in the corroded zone (Spectrum 1) with respect to the un-corroded zones (Spectrum 2).

### 3.3. Constant Loading (CL) Tests

**Figure 10:**
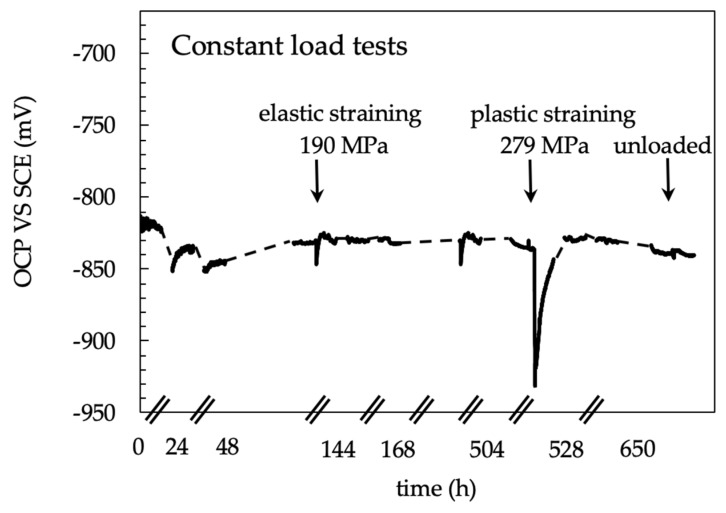
Open circuit potential measurements of the specimen loaded at different values during CL test. The decrease in the OCP mainly occurs as loading increase due to aluminum corrosion products cracking.

**Figure 11:**
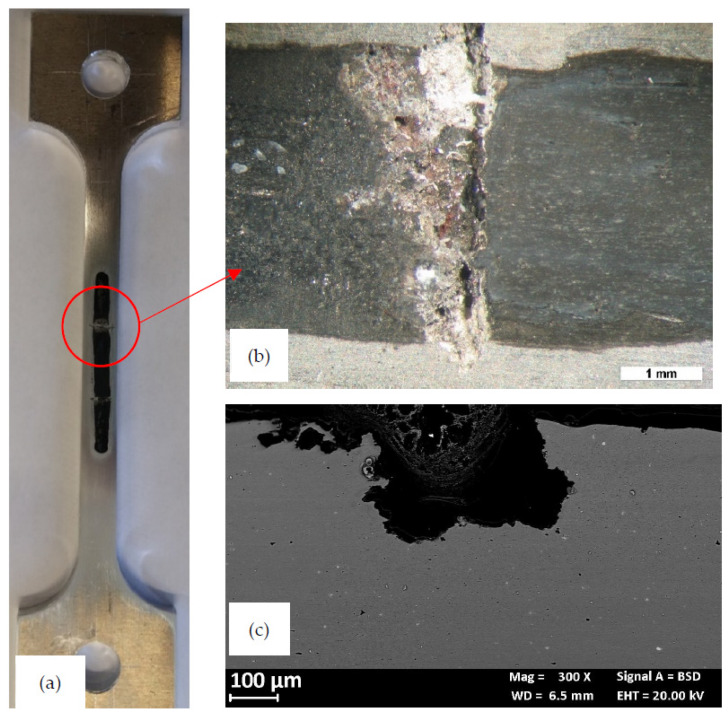
Aspect of the specimen at the end of the constant load test. (**a**) Macro image: the dark zone in the central part exposed to the NaCl 0.6 M solution; (**b**) close-up of the red circle with the selective attack of the TMAZ; (**c**) metallographic section of localized attack.

**Figure 12:**
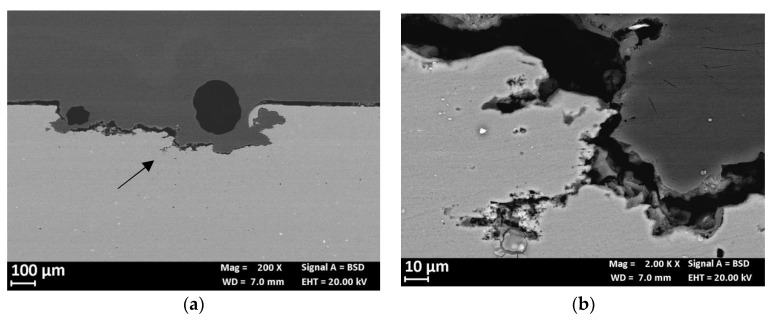
(**a**) Metallographic section of the enlarged localized attack in the TMAZ zone of the CL specimen. Close-up of the arrow in (**b**) in which is evident the change the morphology of attack that allow the rolling direction.

Figure 10 shows the effect of the applied load on the OCP of the CL specimen. The OCP of the unloaded specimens rapidly stabilized between −850 and −800 mV vs SCE. The fluctuations are mainly ascribable to the removal of corrosion products of aluminum formed at very early exposures, due to the recirculation of the testing solution. When the specimen is loaded in elastic field, a decrease in the OCP was noticed but the potential values stayed in the same range measured in unloaded condition. Conversely, at strain level exceeding the yield stress—i.e., in the plastic field—100 mV decrease in the OCP occurred due both to the rupture of the thick corrosion product scale of aluminum and the plastic straining exposes the very active metal to the aggressive environment. The OCP came back to the initial value after about 24 h. This value remained almost constant, until the end of the tests. The specimen did not break during the tests but showed a corrosion attack mainly localized at the HAZ (Figure 11). Differently from the 4PBB specimens, the attack appears wider, and positioned perpendicularly to the loading direction coinciding with the rolling direction (Figure 11c and Figure 12a). Some small ramifications along the grain boundaries were observed (Figure 12b).

### 3.4. Slow Strain-Rate (SSR) Tests

**Figure 13:**
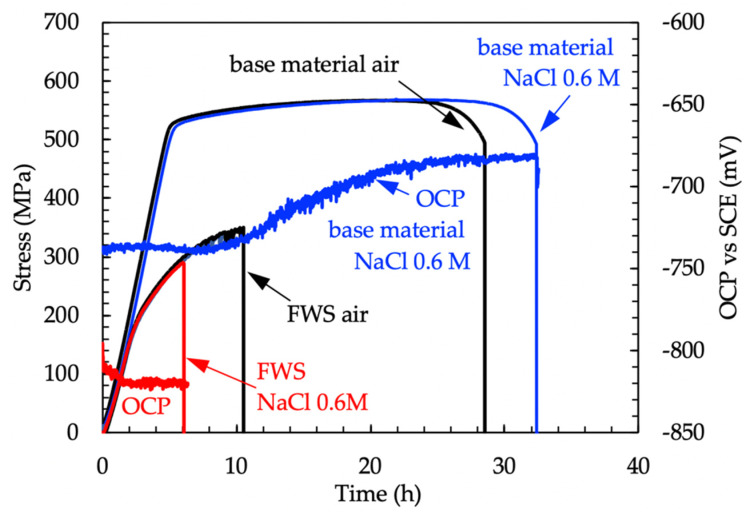
Stress vs time and OCP of the specimens during the slow strain-rate tests.

Figure 13 reports the load and OCP measurements performed during the SSR tests of the AA 7075-T6 base materials and the FSW joints. The tests were twice repeated, but only one curve for each condition is reported in the graph for simplicity. The tensile curves of the base material and the weld obtained in air or in NaCl 0.6 M are practically overlapped. The time to failure of the base material is close to the mean value with a very small deviation of 7%. 25% deviation was observed for the welded specimens. No stress-corrosion crack occurrence was noticed for the base metal and the time to failure in 0.6 M NaCl solution is even longer compared to the value measured at air. The role of active corrosion in NaCl solution can be hypothesized to enhance dislocation mobility and thus the plastic deformation, as reported by Jones et al. [28,29]. The authors named this phenomenon anodic attenuation of strain hardening.

**Figure 14:**
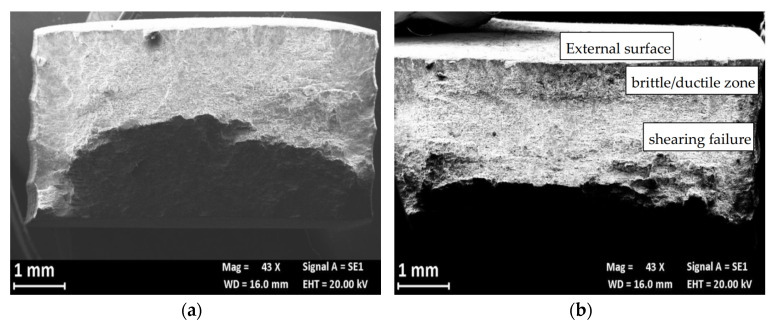
SEM image of the fracture surface of the AA7075 T6 specimen after the SSR test (**a**) macro at air, (**b**) macro in 0.6 M NaCl solution.

**Figure 15:**
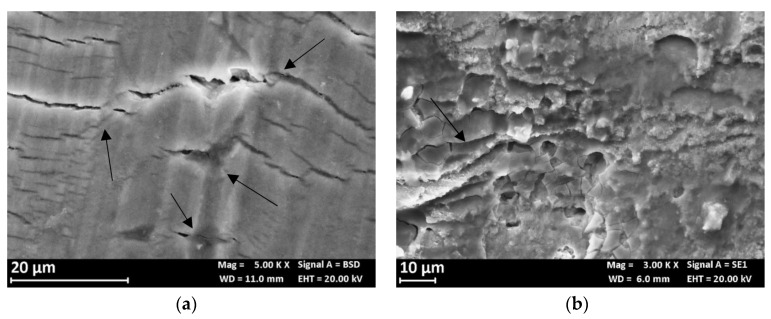
SEM image of the fracture surface of the AA7075 T6 specimen after the SSR test (**a**) specimen surface with corrosion products of aluminum and several microcracks at macro-precipitates (black arrows); (**b**) particular of fracture initiation zone after SSR test in 0.6 M NaCl solution.

**Figure 16:**
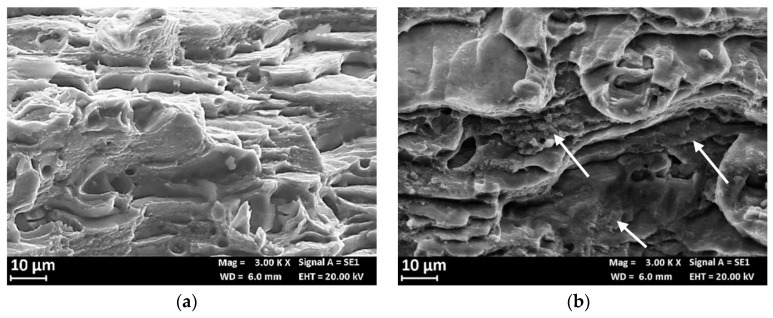
SEM image of the fracture surface of the AA7075 T6 specimen after the SSR test: (**a**) particular of the fracture surface at air and (**b**) in 0.6M NaCl solution (the arrows indicate small areas with typical SCC morphology).

Figure 14 compares the fracture surface of the SSR specimens after the test in air and in 0.6 M NaCl solution. The fracture surface at air (Figure 14a) showed a shearing failure, typical of the prismatic specimens, while the fracture surface of the specimen after the SSR test in 0.6 M NaCl solution exhibits an initial flat area (Figure 14b) due to the presence of microdefects able to trigger localized corrosion initiation. These microcracks are mainly at macro-precipitates (black arrows in Figure 15a) and have depth less than 100 μm. In the correspondence of these microcracks, the fracture surface is heavily corroded (Figure 15b) and it is possible to observe the presence of several micro-precipitates. The flat zone of the fracture surface (Figure 16b) is mixed brittle/ductile as some small dimples and quasi cleavage areas were observed, similarly to the fracture surface at air (Figure 16a). The presence of very small zones with typical SCC morphology, indicated with the arrows in Figure 16b [29] was also confirmed; the final shearing fracture of these specimens was similar to that in air.

**Figure 17:**
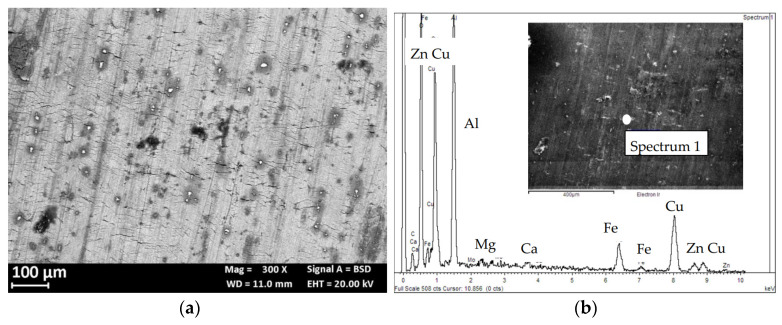
(**a**) Backscatter electron detector (BSD) image of the external surface of a base material specimen after the SSR test in NaCl 0.6 M, the bright zone indicates precipitates with higher atomic weight than the aluminum matrix; (**b**) EDX spectrum of one of these particles.

No macroscopic SCC phenomena were observed probably due to the strain rate (10^−6^ s^−1^) value adopted and very aggressive NaCl solution. Results presented in other works [30] confirmed the absence of stress corrosion on this alloy during the SSR tests at the OCP and strain rate of 10^−6^ s^−1^. The OCP remains constant in the elastic field, but it increases with the plastic deformation, probably due to the enrichment in iron and copper due to the dissolution of the surrounding aluminum matrix. This observation is supported by back-scattered electron (BSE) image and EDX spectrum (Figure 17).

The curves of the FSW joints exhibit time-to-failure values lower than the base material. This can be mainly ascribable to the decrease in the tensile properties at the joint TMAZ/HAZ zone, as observed also elsewhere [26,31,32,33]. Consequently, the hardness and then the UTS of the alloy decrease due to microstructural modifications [34,35].

**Figure 18:**
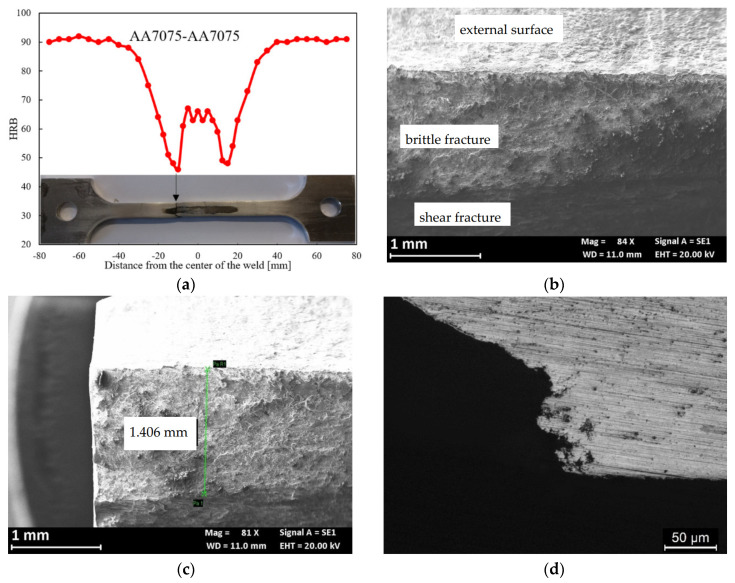
(**a**) Hardness profile along the FSW butt joint; (**b**) SEM image of the fracture surface, a flat zone of about 1 mm of growth (close-up in (**c**) is highlighted in (**d**)) metallographic section of the fracture surface in which is visible the flat zone and the small ramifications.

**Figure 19:**
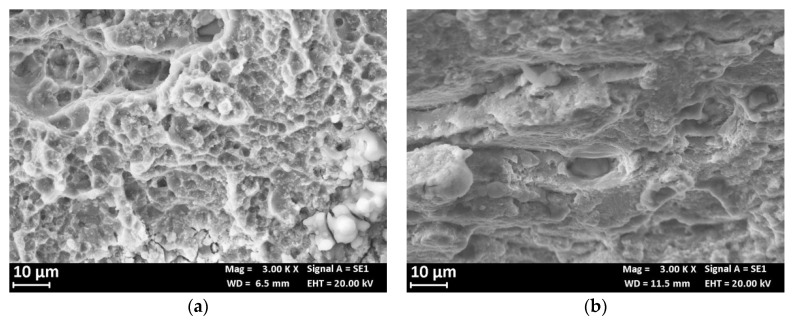
(**a**) Particular of the mixed ductile/brittle fracture zone and (**b**) of the ductile shearing final rupture.

**Figure 20:**
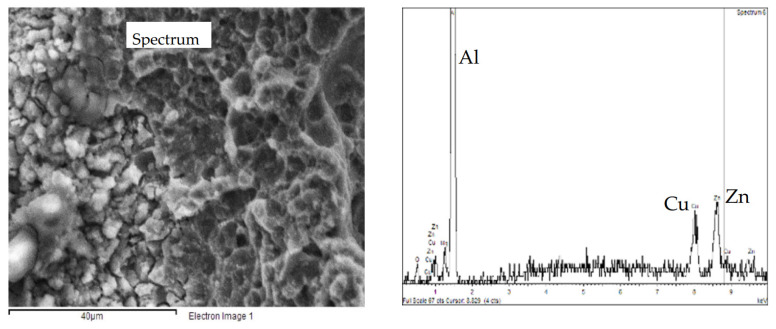
EDS spectrum of the precipitates present on the mixed ductile/brittle zone of the fracture surface of the FWS specimen after the SSR test in 0.6 M NaCl solution.

Figure 18a reports the hardness profile of the longitudinal section of the weld. The hardness sharply decreases at the TMAZ/HAZ and, therefore, the plastic deformation mainly occurs at this point, thus decreasing the total elongation at break and the time of failure. Despite the high strain rate due to plastic strain localization at TMAZ/HAZ, slight decrease in the time to failure in 0.6 M NaCl solution was noticed compared to air—i.e., 10 ± 0.5 h—thus denoting SCC occurrence. Bala Srinivasana et al. reported about SCC phenomena in the HAZ of the AA7075 of mixed FWS joints of AA7075 and AA6056, when the specimens were tested at strain rate 10^−7^ s^−1^ in NaCl solutions, but only localized corrosion in the tests carried out at 10^−6^ s^−1^ was detected [36]. The SEM observation of the surfaces demonstrate a flat zone at fracture initiation with maximum depth of 1 mm (Figure 18b,c). The presence of non-coherent corrosion products of aluminum and several precipitates was also noticed (Figure 19a). EDX analysis detected the presence of zinc, aluminum, and copper, as well as magnesium depletion (Figure 20). The final fracture is by shearing (Figure 18b) and its morphology is characterized by elongated dimples and a few brittle areas Figure 19b), similarly to the base material (Figure 16).

The OCP of the FSW butt joints lies in the range of −820–−810 mV vs SCE and it is less noble than the base material, whose potential was in the range between −725 and −670 mV vs SCE. The OCP of the FSW alloy decreases during the elastic deformation. Conversely, constant values were detected once the specimen was strained in the plastic field. The values of the OCP of the FSW specimens during SSR tests are close to that measured during 4PBB tests and CL tests (Figure 7 and Figure 10), corresponding to the OCP of the TMAZ/HAZ and the nugget (Figure 8). The very low OCP potential indicates that these zones are more susceptible to corrosion than the base material, as confirmed by the intense attack observed on the specimens at the end of the test.

## 4. Discussion

These results confirmed the higher corrosion susceptibility of the TMAZ/HAZ of the AA 7075 FSW joints with respect to the nugget and the base material, but there is no agreement between the different geometries of the specimens.

No SCC occurrence was noticed after 4PBB tests, but severe localized attack along the rolling direction at TMAZ/HAZ. Shallow defects at TMAZ/HAZ were observed after CL and SSR tests, which direction was perpendicular respect to the applied load. 

The higher corrosion susceptibility of the TMAZ/HAZ can be attributed to the microstructure modification produced by the FSW process. The presence of precipitates at the grain boundaries less noble than the matrix promotes the intergranular corrosion of the alloy [37]. Different mechanisms were proposed to explain their effect, Maitra and English affirmed that the grain boundaries have a more active breakdown than the matrix and preferentially initiated the pitting [38], while other authors consider the effect of acidification generated by the preferential dissolution of the anodic particles as the cause of the continuous grain-boundary attack [11,14]. The intergranular attack through thickness direction (short transverse—S) is much lower than that in either longitudinal (L) and transverse (T) directions for rolled plates owing to the microstructural anisotropy while the breakdown potentials were independent of sample orientations [39].

Similar mechanisms have been formulated to explain the SCC mechanism of aluminum 7XXX alloys, mainly hypothesizing hydrogen embrittlement [18,40], but also slip-dissolution mechanism [41]. Naijar et al. [42] proposed a mixed mechanism, in which the SCC phenomena were initiated from localized attacks at macro-precipitates present on the alloy surface (i.e., Al_7_Cu_2_Fe or Al_2_CuMg) or on grain boundaries. The hydrolysis of the produced cations induces the local acidification of the occluded cell and, as consequence, the possibility to electrochemical evolution of hydrogen. These authors suggested that localized dissolution at precipitates or at the grain boundary correspond to the formation of critical defects, which is the first step of the SCC. The deterioration of the passive film and the anodic dissolution of the metal promotes hydrogen uptake and diffusion inside the metal lattice. As the defects act as local stress intensifier, intergranular fracture are promoted, especially in the short-transverse direction for which grain boundaries are favorably oriented to the applied tensile stress [43,44]. Vasudevan and Sadananda [45] reported the decrease in the crack propagation rate in NaCl solution with the increasing of the precipitates size at the grain boundaries, and the depletion in copper of the aluminum matrix. The positive effect of precipitates size was also underlined by other authors [46].

On the contrary, the results of Ashai et al. [47] on smooth specimens of Al-4.3Zn-1.7Mg alloys in 3.5% NaCl + 0.2% H_2_O_2_ solution, demonstrated an inverse correlation between the time to failure (failure rate) and the size of precipitates at the grain boundaries. This can be ascribed to the preferential dissolution of the MgZn_2_ precipitates at the grain boundaries [48].

Talianker and Cina demonstrated a clear relationship between the presence of dislocations aside to grain boundaries and the susceptibility to stress corrosion of AA7XXX. [48].

The fractographic analysis of the specimens demonstrated the microcracks initiation preferentially at larger precipitates. The nugget and the TMAZ/HAZ showed lower OCP, thus indicating higher susceptibility to corrosion than the base material. No SCC microcracks propagation was noticed at the nugget due to the large size of the strengthening MgZn_2_ micro-precipitates—that decreased the SCC susceptibility—and the higher tensile strength compared to the TMAZ/HAZ zones. Under constant deformation tests, such as 4PBB tests, the applied stress naturally decreases during the exposure as localized attack propagate toward the specimen thickness. In addition, the tensile stress acts on these specimens in the direction of the elongated grains, and therefore the SCC cracks should propagate in the perpendicular direction. For these reasons, corrosion takes place in form of localized attacks at the elongated grain boundaries along the rolling direction (Figure 9). 

In SSR and CL tests, the localized attacks and the plastic strain mainly occurred at TMAZ/HAZ, thus triggering the formation of SCC crack embryos. Based on the abovementioned mechanism, hydrogen embrittlement should promote SCC microcracks propagation. Hydrogen supplied by both the cathodic reaction and the anodic dissolution of the matrix/precipitate interphase can diffuse in the high stressed zone and promotes brittle crack propagation. The SEM analysis confirms the presence of several submicrometric precipitates in the correspondence of the brittle areas of the fracture surface. The iron-rich phases can consequently enhance the hydrogen evolution mechanism, because the hydrogen overpotential on these phases is lower than that on aluminum. Conversely, the applied plastic strain enhanced the corrosion rate by means of the chemomechanical effect underlined by Gutman. This effect is higher at the interface between the matrix and second phases, which acts as an obstacle to the movement of the dislocations [28,49]. In these zones, the very high dissolution rate of the alloy is prevalent, and hydrogen cannot reach the critical concentration to produce cracking. In the CL tests the attack becomes enlarged and no failure of the specimens were observed. On the other hand, the only small propagation of microcracks noticed after SSR tests suggests that the final rupture quickly occurred with a shear mechanism because of the high strain rate at the TMAZ/HAZ. For this reason, no evident stress-corrosion cracking phenomena were observed. 

## 5. Conclusions

The paper studied the stress-corrosion cracking behavior of AA7075 T6 aluminum joints made by FSW as a function of loading conditions. SCC tests were performed by considering constant deformation (4PBB and CL) conditions and SSR conditions in aerated NaCl 35g/L solution at room temperature.

Certain susceptibility to SCC was demonstrated for AA 7075 FSW butt joints tested in this work mainly at the TMAZ/HAZ as nucleation of crack embryos occurred during both CL and SSR tests. However, high dissolution rate of the metal inside the localized attack prevents the achievement of critical crack size to promote its propagation up to rupture.

Slight decrease in the free corrosion potential was noticed because of the modification of the microstructure introduced by the welding process mainly at the TMAZ/HAZ and the nugget with respect the base material. As the TMAZ/HAZ zone is characterized by low tensile strength compared to both the nugget and the base material, it is preferentially strained under loading conditions. The plastic strain then enhances the active dissolution rate of the metal matrix and severe attack takes place, thus hindering SCC crack propagation. 

The penetration depth of the intergranular attack was deeper after the four-point bent-beam tests compared to all the other testing techniques. Preferential dissolution along the grain boundaries was found in heat-affected zones and the attack follows the elongated grain structure along the rolling direction.

## Figures and Tables

**Table 1:** Chemical composition (% weight) of the alloy 7075-T6.

Al	Si	Fe	Cu	Mn	Mg	Zn	Ti	Cr
bulk	0.05	0.10	1.53	0.008	2.54%	5.72%	0.04	0.20

**Table 2:** Mechanical properties of the base materials and the welded joint.

Base Material			FWS Joint		
YS (MPa)	UTS (MPa)	Max Strain (%)	YS (MPa)	UTS (MPa)	Max Strain (%)
512	576	13.5	-	329	11
